# Prognostic Impact of Periprocedural Myocardial Infarction in Patients with Heavily Calcified Coronary Artery Disease Receiving Rotational Atherectomy

**DOI:** 10.31083/j.rcm2402042

**Published:** 2023-02-02

**Authors:** Jin Jung, Sung-Ho Her, Kyusup Lee, Ki-Dong Yoo, Keon-Woong Moon, Donggyu Moon, Su Nam Lee, Won Young Jang, Ik Jun Choi, Jae-Hwan Lee, Jang Hoon Lee, Sang Rok Lee, Seung-Whan Lee, Kyeong Ho Yun, Hyun-Jong Lee

**Affiliations:** ^1^Department of Cardiology, St. Vincent’s Hospital, College of Medicine, The Catholic University of Korea, 16247 Seoul, Republic of Korea; ^2^Department of Cardiology, Daejeon St. Mary’s Hospital, College of Medicine, The Catholic University of Korea, 34943 Seoul, Republic of Korea; ^3^Department of Cardiology, Incheon St. Mary’s Hospital, College of Medicine, The Catholic University of Korea, 21431 Incheon, Republic of Korea; ^4^Department of Cardiology in Internal Medicine, Chungnam National University School of Medicine, Chungnam National University Sejong Hospital, 30099 Sejong, Republic of Korea; ^5^Department of Internal Medicine, Kyungpook National University Hospital, School of Medicine, Kyungpook National University, 41944 Daegu, Republic of Korea; ^6^Department of Cardiology, Chonbuk National University Hospital, 54907 Jeonju, Republic of Korea; ^7^Department of Cardiology, Asan Medical Center, University of Ulsan College of Medicine, 05505 Seoul, Republic of Korea; ^8^Department of Cardiovascular Medicine, Regional Cardiocerebrovascular Center, Wonkwang University Hospital, 54538 Iksan, Republic of Korea; ^9^Department of Internal Medicine, Sejong General Hospital, 14754 Bucheon, Republic of Korea

**Keywords:** periprocedural myocardial infarction, coronary artery calcification, rotational atherectomy, clinical outcome

## Abstract

**Background:**

Periprocedural myocardial infarction (PMI) occurs more frequently in patients with heavily calcified lesion and undergoing rotational atherectomy (RA). However, there are limited studies addressing prognostic impact of PMI in patients requiring RA due to severe coronary artery calcification (CAC). Therefore, the objective of this study was to determine the prognostic impact of PMI in patients who underwent percutaneous coronary intervention (PCI) using RA.

**Methods:**

A total of 540 patients (583 lesions) who received PCI using RA were enrolled between January 2010 and October 2019. PMI was defined as elevations of creatine kinase-myocardial band (CK-MB) >10 times the upper limited normal. Patients were divided into a PMI group and a non-PMI group. Primary endpoint was major adverse cardiovascular and cerebrovascular event (MACCE), a composite of cardiac death, target-vessel myocardial infarction, target-vessel revascularization, and cerebrovascular accident.

**Results:**

Although in-hospital events occurred more frequently in the PMI group than in the non-PMI group (15 [3.0%] vs. 6 [13.3%], *p* = 0.005), the incidence of MACCEs at 1 month, 1–12 months, or 12 months failed to show a significant difference between the two groups (1 month, 10 [2.0%] vs. 1 [2.2%], *p *> 0.999; 1–12 months, 39 [7.9%] vs. 7 [15.6%], *p* = 0.091; 12 months, 49 [9.9%] vs. 8 [17.8%], *p* = 0.123).

**Conclusions:**

This study shows that PMI after RA in patients with severe CAC was associated with more frequent in-hospital events and a nonsignificant trend for more events during 1 year follow-up.

## 1. Introduction

Technological advances in coronary intervention over the past four decades have made safer percutaneous coronary intervention (PCI) possible, with both clinical outcomes and procedural complications showing significant improvement. Although the incidence of periprocedural myocardial infarction (PMI) widely varies according to the definition, biomarker, biomarker threshold, and clinical presentation, it still remains one of the most common complication [1, 2].

PMI, referred to as myocardial injury that occurs during revascularization procedures [3], occurs more frequently in patients with heavily calcified lesion [4] and those who undergo rotational atherectomy (RA) for modifying that lesion [5, 6, 7]. Therefore, PMI is an important issue in patients requiring RA due to severe coronary artery calcification (CAC).

Although several previous studies have investigated the prognostic impact of PMI in patients undergoing PCI, results are still under debate [1, 6, 8, 9, 10, 11, 12, 13]. As mentioned above, although PMI is a major problem in patients requiring RA due to severe CAC, few studies have reported the prognostic impact of PMI in those patients. Therefore, the objective of the present study was to determine the prognostic impact of PMI on clinical outcomes of patients who underwent PCI using RA.

## 2. Materials and Methods

The study population consisted of 540 patients (583 lesions) with severely calcified coronary artery disease (CAD) who underwent PCI using RA from January 2010 to October 2019 at nine tertiary centers in Korea. Patients were included within the ‘ROtational atherectomy in Calcified lesions in Korea (ROCK)’ registry. This registry was approved by the Institutional Review Board (IRB) of each hospital. Data were collected at each center using a standardized case report form to record clinical and demographic characteristics, procedure related data, and follow-up data. Follow-up data were obtained up to 12 months retrospectively based on patients’ medical records and/or telephone interviews conducted by research nurses.

**Fig. 1.**
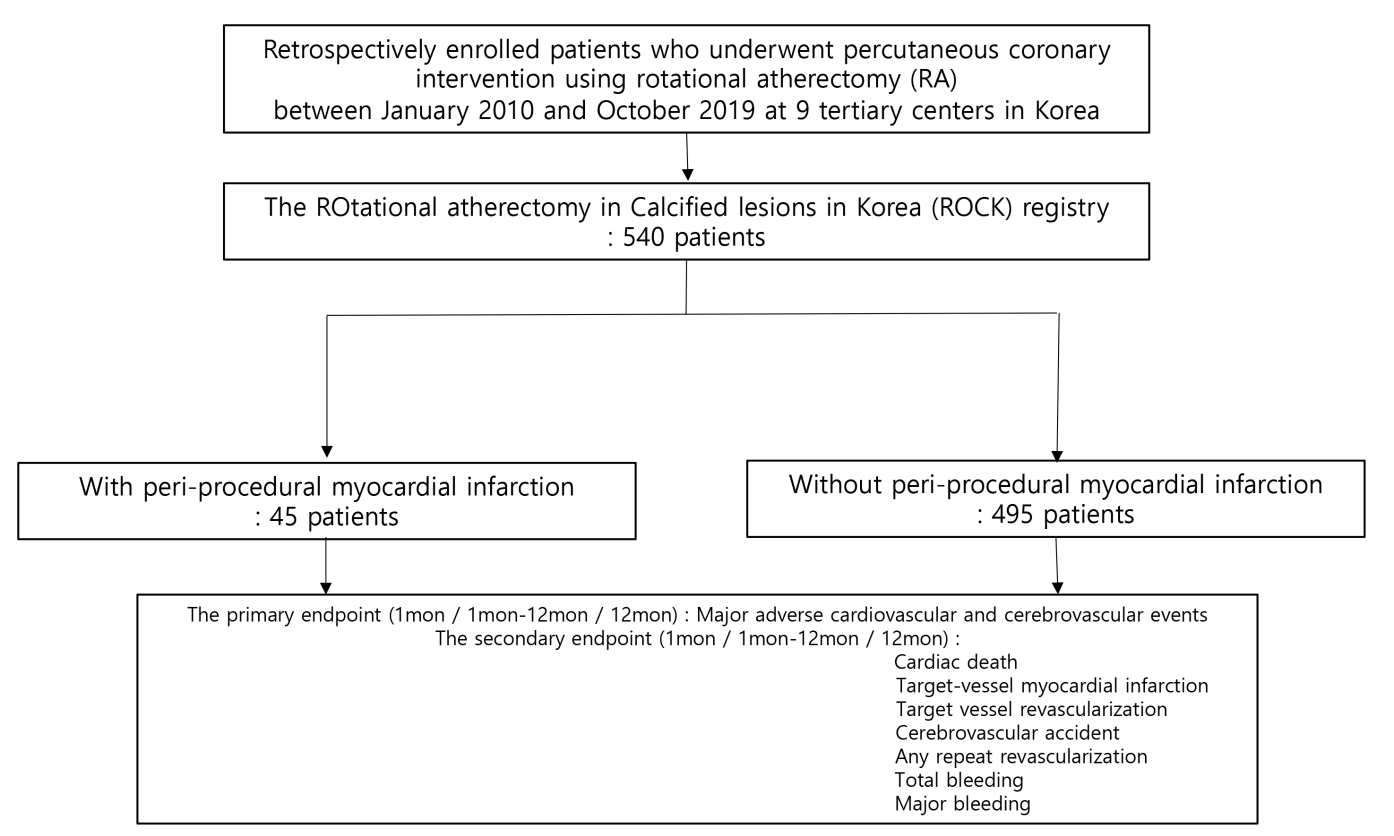
**Study population flow chart**.

Patients were divided into two subgroups based on the presence or absence of PMI. The flow chart is displayed in Fig. 1. Baseline characteristics and clinical outcomes were compared between the two groups.

All RA procedures were performed using a ${\mathrm{Rotablator}}^{\text{TM}}$ RA system (Boston Scientific, Marlborough, MA, USA) guided by standard techniques. Procedure related treatment strategy was dependent on discretion of attending operators. Patients’ management including medical treatment was performed in accordance with accepted guidelines and established standard care [14].

PMI was defined with reference to Society for Cardiovascular Angiography and Interventions (SCAI) definition [15]. In patients with normal baseline creatine kinase-myocardial band (CK-MB), PMI was defined as peak elevation of CK-MB ≥10× upper limited normal (ULN) within 48 hours of the procedure. In patients with elevated baseline CK-MB, PMI was defined a new CK-MB elevation by an absolute increment of ≥10× ULN from the previous nadir level. The primary endpoint was the occurrence of major adverse cardiovascular and cerebrovascular events (MACCEs) defined as a composite outcome of cardiac death, target-vessel spontaneous myocardial infarction (TVMI), target-vessel revascularization (TVR), or cerebrovascular accident (CVA). Secondary endpoints were cardiac death, TVMI, any repeat revascularization (RR), TVR, CVA, and bleeding. In-hospital events and procedural outcomes were also investigated. These definitions were the same as previously published report [16]. Chronic kidney disease (CKD) was defined as an estimated glomerular filtration rate <60 mL/min/1.73 $m^{2}$ as calculated using the Modification of Renal Diet (MDRD) equation from baseline serum creatinine [17]. All clinical events were confirmed by source documentation collected at each enrolled hospital and centrally adjudicated by an independent group of clinicians unaware of the revascularization type.

Continuous variables are presented as median and interquartile range or mean ± standard deviation using Student’s *t*-test. Categorical variables are expressed as numbers and percentages. Differences between two groups were compared using chi-square test or Fisher’s exact test. Univariable and multivariable Cox regression analyses were performed. Hazard ratio (HR) and 95% confidence interval (CI) were also calculated. For multivariate analysis, confounding factors were age, sex, body mass index (BMI), clinical diagnosis, coronary perforation, coronary dissection, left ventricle ejection fraction (LVEF), and procedural success. Event rates were estimated using Kaplan–Meier estimates in time-to-first-event analyses and compared using the log-rank test. A *p*-value < 0.05 was considered statistically significant. All statistical analyses were performed using Statistical Analysis Software (SAS) version 9.2 (SAS Institute, Cary, NC, USA).

## 3. Results

### 3.1 Baseline Characteristics

Patients were divided into a PMI group and a non-PMI group according to the occurrence of PMI. Among a total of 540 patients, 45 patients were classified into the PMI group and the remaining 495 patients were classified into the non-PMI group. Baseline characteristics of patients with and without PMI are presented in Table 1 and Table 2, respectively. Procedural details are also presented in Table 2. There was no significant difference in baseline characteristics between the two groups except for BMI, clinical diagnosis, and LVEF. Especially, left main (LM) disease, mean stent diameter, total number of stents, and stent length did not show any significant difference between non-PMI and PMI groups in this study (LM disease, 68 [13.7%] vs. 6 [13.3%], *p* = 0.940; mean stent diameter, 3.0 ± 0.4 vs. 3.0 ± 0.3, *p* = 0.895; total number of stents, 2.3 ± 1.1 vs. 2.5 ± 1.2, *p* = 0.370; total stent length, 66.6 ± 34.4 vs. 66.7 ± 32.3, *p* = 0.990). On the other hand, acute coronary syndrome (ACS) was higher in the PMI group (37 [82.2%] vs. 291 [58.8%], *p* = 0.002).

**Table 1.** **Baseline demographic and clinical characteristics**.

		Non-PMI (n = 495)	PMI (n = 45)	*p*-value
Age, years		71.2 ± 10.2	73.6 ± 8.5	0.129
Sex				0.979
	Male	296 (59.8)	27 (60.0)	
	Female	199 (40.2)	18 (40.0)	
Smoking		93 (18.8)	10 (22.2)	0.575
BMI		24.3 ± 3.9	22.8 ± 3.5	0.009
HTN		380 (76.8)	35 (77.8)	0.878
Hyperlipidemia		215 (43.4)	20 (44.4)	0.896
DM		285 (57.6)	20 (44.4)	0.089
CKD		88 (17.8)	8 (17.8)	>0.999
Dialysis		46 (9.3)	3 (6.7)	0.787
Previous PCI		129 (26.1)	10 (22.2)	0.573
Previous CABG		22 (4.4)	2 (4.4)	>0.999
Previous MI		63 (12.7)	3 (6.7)	0.235
CVA		66 (13.3)	9 (20.0)	0.216
PVD		37 (7.5)	2 (4.4)	0.761
Chronic lung disease		33 (6.7)	4 (8.9)	0.536
Heart failure		67 (13.5)	10 (22.2)	0.111
Atrial fibrillation		43 (8.7)	6 (13.3)	0.281
Clinical diagnosis				0.002
	Stable angina	204 (41.2)	8 (17.8)	
	ACS	291 (58.8)	37 (82.2)	
HbA1C		6.7 ± 1.5	6.2 ± 1.1	0.084
Total cholesterol		143.2 ± 38.5	148.3 ± 41.1	0.419
LDL cholesterol		84.6 ± 39.6	86.4 ± 37.4	0.798
HDL cholesterol		46.0 ± 14.5	46.7 ± 14.5	0.791
Triglyceride		121.2 ± 76.1	100.1 ± 34.8	0.110

Data are shown as mean ± SD or n (%). PMI, periprocedural myocardial infarction; BMI, body mass index; HTN, hypertension; CKD, chronic kidney disease; DM, diabetes mellitus; PCI, percutaneous coronary intervention; CABG, coronary artery bypass graft; MI, myocardial infarction; CVA, cerebrovascular accident; PVD, peripheral vascular disease; ACS, acute coronary syndrome; HbA1c, glycated hemoglobin; LDL, low density lipoprotein cholesterol; HDL, high density lipoprotein cholesterol.

**Table 2.** **Baseline angiographic characteristics and procedural details**.

		Non-PMI (n = 495)	PMI (n = 45)	*p*-value
ACC/AHA classification				0.670
	A	3 (0.6)	0 (0.0)	
	B1	38 (7.3)	2 (4.4)	
	B2	49 (9.9)	3 (6.7)	
	C	405 (81.8)	40 (88.9)	
Left main disease		68 (13.7)	6 (13.3)	0.940
MVD		385 (77.8)	39 (86.7)	0.195
IVUS		231 (46.7)	18 (40.0)	0.390
Arc of calficication >270°		91/150 (60.7)	10/18 (55.6)	0.676
LVEF, %		53.5 ± 13.0	47.5 ± 16.3	0.004
Mean stent diameter, mm		3.0 ± 0.4	3.0 ± 0.3	0.895
Total number of stent		2.3 ± 1.1	2.5 ± 1.2	0.370
Total stent length, mm		66.6 ± 34.4	66.7 ± 32.3	0.990
Procedure time, min		79.0 ± 51.8	81.8 ± 35.7	0.728

Data are shown as mean ± SD or n (%) or n/N (%). PMI, periprocedural myocardial infarction; ACC/AHA, American College of Cardiology/American Heart Association; MVD, multivessel disease; IVUS, intravascular ultrasound; LVEF, left ventricle ejection fraction.

### 3.2 In-Hospital Events and Procedural Outcomes

Compared with the non-PMI group, the PMI group showed more frequent in-hospital events (6 [13.3%] vs. 15 [3.0%], *p* = 0.005), coronary dissection (8 [17.8%] vs. 38 [7.7%], *p* = 0.043), and coronary perforation (3 [6.7%] vs. 7 [1.4%], *p* = 0.043) (Table 3). Coronary dissection and coronary perforation are among mechanisms of PMI.

**Table 3.** **In-hospital events and procedural outcomes**.

		Non-PMI (n = 495)	PMI (n = 45)	*p*-value
In-hospital events		15 (3.0)	6 (13.3)	0.005
In-hospital death		8 (1.6)	3 (6.7)	0.056
Urgent CABG		2 (0.4)	0 (0.0)	>0.999
Urgent PCI		5 (1.0)	2 (4.4)	0.109
In-hospital CVA		1 (0.2)	1 (2.2)	0.160
Procedural outcomes				
	Coronary dissection*	38 (7.7)	8 (17.8)	0.043
	Temporary pacemaker during procedure	15 (3.0)	1 (2.2)	>0.999
	Coronary perforation	7 (1.4)	3 (6.7)	0.043
	In-hospital bleeding	22 (4.4)	5 (11.1)	0.064
	Procedure success	483 (97.6)	37 (82.2)	<0.001

Data are shown as mean ± SD or n (%). *Coronary dissection from defined from The National Heart, Lung, and Blood Institute (NHLBI) classification system. PMI, periprocedural myocardial infarction; CABG, coronary artery bypass grafting; PCI, percutaneous coronary intervention; CVA, cerebrovascular accident.

### 3.3 Clinical Outcomes

**Fig. 2.**
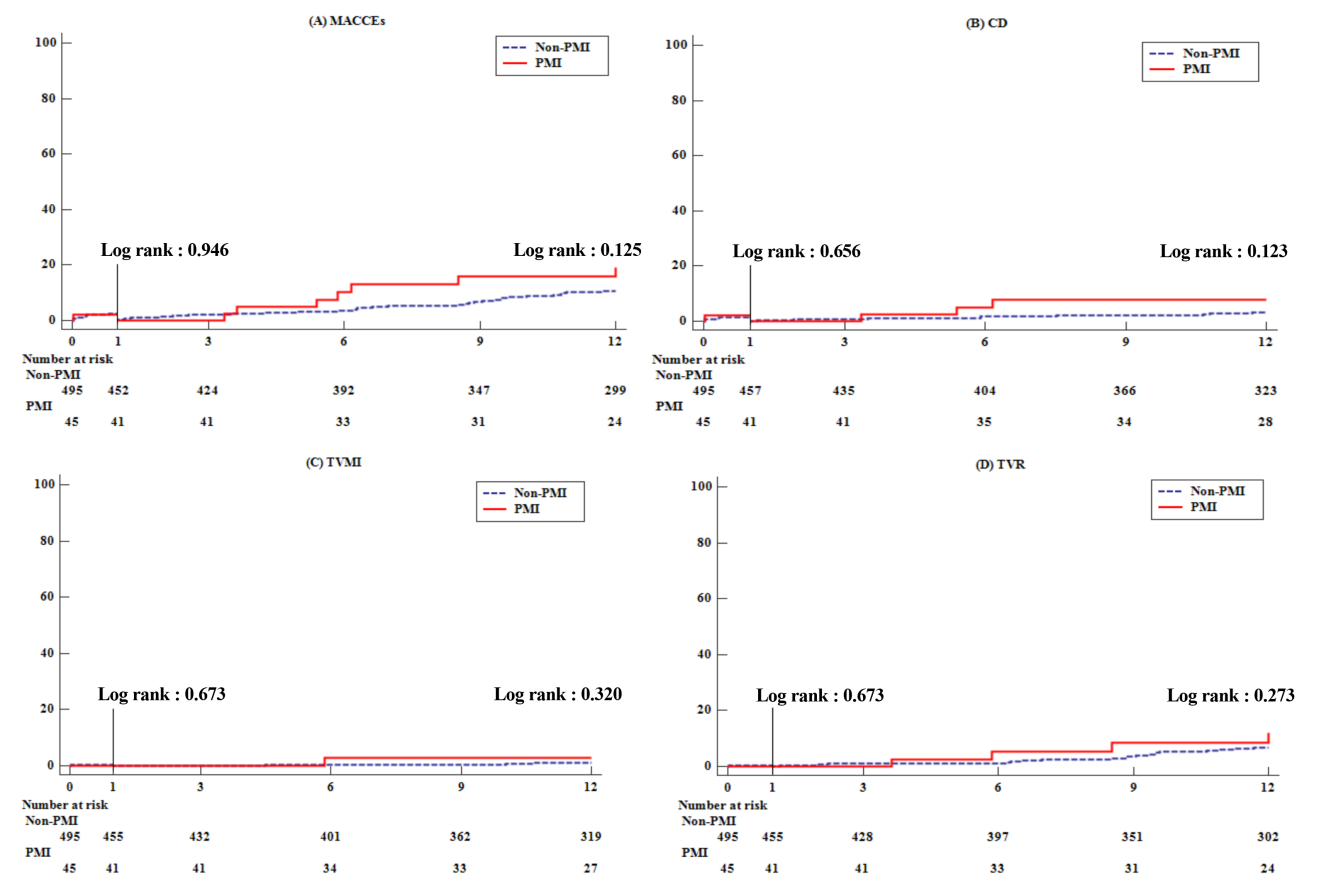
**Kaplan-meier curve for clinical outcomes during follow-up**. (A) Major cardiovascular and cerebrovascular events. (B) Cardiac death. (C) Target vessel myocardial infarction. (D) Target vessel revascularization.

The incidence of MACCE, the primary endpoint, showed no significant difference between the two groups at 1 month, 1–12 months, or 12 months. There was no significant difference in secondary endpoints such as cardiac death, target-vessel MI, TVR, or CVA. Only total bleeding at 1 month showed a tendency to occur more frequently in the PMI group (Fig. 2) (Table 4).

**Table 4.** **Clinical outcomes and univariable/multivariable cox regression analysis**.

		Non-PMI (n = 495)	PMI (n = 45)	*p*-value	Univariate HR (95% CI)	*p*-value	Multivariate HR** (95% CI)	*p*-value
Endpoints at 1 month								
	MACCEs	10 (2.0)	1 (2.2)	>0.999	0.933 (0.121–7.172)	0.947	0.298 (0.029–3.015)	0.305
	Cardiac death	7 (1.4)	1 (2.2)	0.504	1.602 (0.197–13.023)	0.659	0.794 (0.062–10.148)	0.859
	Target-vessel MI	2 (0.4)	0 (0.0)	>0.999	-	-	-	-
	TVR	2 (0.4)	0 (0.0)	>0.999	-	-	-	-
	CVA	2 (0.4)	0 (0.0)	>0.999	-	-	-	-
	Any repeat revascularization	2 (0.4)	0 (0.0)	>0.999	-	-	-	-
	Total bleeding	3 (0.6)	4 (8.9)	0.001	15.037 (3.365–67.193)	0.000	8.464 (1.350–53.043)	0.023
	Major bleeding	1 (0.2)	1 (2.2)	0.160	11.121 (0.696–177.807)	0.089	41.075 (0.042–40365)	0.291
Endpoints at 1 month–12 month								
	MACCEs	39 (7.9)	7 (15.6)	0.091	1.852 (0.833–4.117)	0.131	1.714 (0.727–4.039)	0.218
	Cardiac death	13 (2.6)	3 (6.7)	0.141	2.591 (0.738–9.092)	0.137	1.349 (0.345–5.272)	0.667
	Target-vessel MI	4 (0.8)	1 (2.2)	0.354	2.890 (0.323–25.864)	0.343	1.441 (0.138–15.080)	0.760
	TVR	26 (5.3)	4 (8.9)	0.302	1.787 (0.624–5.121)	0.280	1.785 (0.568–5.612)	0.322
	CVA	7 (1.4)	0 (0.0)	>0.999	-	-	-	-
	Any repeat revascularization	30 (6.1)	4 (8.9)	0.514	1.532 (0.540–4.349)	0.423	1.302 (0.417–4.067)	0.650
	Total bleeding	17 (3.4)	1 (2.2)	>0.999	0.679 (0.090–5.103)	0.707	0.491 (0.047–5.186)	0.554
	Major bleeding	5 (1.0)	1 (2.2)	0.408	2.294 (0.268–19.659)	0.448	23.083(0.928–574.0)	0.056
Endpoints at 12 month								
	MACCEs	49 (9.9)	8 (17.8)	0.123	1.680 (0.800–3.531)	0.171	1.314 (0.592–2.913)	0.502
	Cardiac death	20 (4.0)	4 (8.9)	0.130	2.245 (0.767–6.568)	0.140	1.277 (0.398–4.099)	0.681
	Target-vessel MI	6 (1.2)	1 (2.2)	0.458	1.911 (0.230–15.875)	0.549	1.078 (0.115–10.109)	0.947
	TVR	28 (5.7)	4 (8.9)	0.329	1.656 (0.581–4.721)	0.345	1.465 (0.471–4.558)	0.510
	CVA	9 (1.8)	0 (0.0)	>0.999	-	-	-	-
	Any repeat revascularization	32 (6.5)	4 (8.9)	0.528	1.435 (0.507–4.057)	0.496	1.103 (0.357–3.405)	0.865
	Total bleeding	20 (4.0)	5 (11.1)	0.048	2.867 (1.076–7.640)	0.035	2.681 (0.869–8.276)	0.086
	Major bleeding	6 (1.2)	2 (4.4)	0.138	3.797 (0.766–18.814)	0.102	18.956(1.542–233.1)	0.022

** adjusted by age, sex, BMI, clinical diagnosis, coronary perforation, coronary dissection, LVEF, procedural success. Data are shown as mean ± SD or n (%). HR, hazard ratio; CI, confidence interval; PMI, periprocedural myocardial infarction; MACCEs, major adverse cardiovascular and cerebrovascular events; MI, myocardial infarction; TVR, target vessel revascularization; CVA, cerebrovascular accident.

## 4. Discussion

Main findings of this study were as follows: (1) stent diameter, number, length, and LM disease in patients who underwent RA showed no significant difference regardless of PMI. (2) PMI was associated with the occurrence of more in-hospital events. (3) There was no significant difference in MACCE during 1 year follow-up between non-PMI and PMI groups though events trended higher in the PMI group. As patients aged and complex PCI increased, patients with severe coronary calcification also increased. Accordingly, procedures requiring RA was increasing. In the present study, there were many patients in both groups with arc of calficication >270° by evaluating IVUS (91 [60.7%] vs. 10 [55.6%], *p* = 0.676). And severe coronary calcification in angiography, defined as radiopacities noted without cardiac motion before contrast injection generally compromising both sides of the arterial lumen [18], is considered to be an arc of calcification of about 215° in IVUS [18]. Taking this into account, it can be seen that much more patients had severely calcified CAD. However, many cardiologists were hesitant to make an RA decision due to concerns about complexity of rotablator procedures and procedure-related adverse events [19]. PMI was also one of the major procedure-related adverse events. Even in the ROCK registry, in-hospital MACCEs occurred in 10.6%. It was mainly driven by PMI (7.9%) [16].

The reason why PMI was a major problem in patients undergoing PCI using RA was related to its mechanism. Mechanisms of PMI include side branch occlusion (SBO), distal embolization, coronary dissection, and coronary perforation [20]. During RA, an additional protection wire for preventing SBO cannot be used. Disrupted calcified plaque can release micro-debris that can induce microembolization and slow/no reflow, thus increasing the risk of coronary dissection and perforation. All these factors can lead to SBO or distal embolization, resulting in PMI during RA [2, 20]. Therefore, it is important to determine whether PMI affects clinical outcome because interventional cardiologists would hesitate to select RA for heavily calcified lesions if PMI affects clinical outcome considerably.

In previous studies, the prognostic impact of PMI was variable depending on biomarkers and biomarker thresholds applied to the definition of PMI [1, 8, 10, 11, 21]. In our study, CK-MB instead of cardiac troponin (cTn) was used as a biomarker. Its threshold was ≥10× ULN. This was because recent studies did not show prognostic significance of cTn measured post-PCI, whereas CK-MB did show such prognostic significance [10, 22]. In previous studies, CK-MB showed prognostic significance only when it was more than 10 times the ULN [8, 13]. Although the use of biomarker and its threshold are known to have prognostic significance, it is important to note that PMI does not affect clinical outcomes as shown in this study. This result was different from previous studies showing that PMI had a negative impact on clinical outcome [1, 8, 9, 13, 21, 23]. In the present study, more than 60% were ACS, whereas Zeitouni M* et al*. [9] was performed only on patients who underwent elective PCI and Lee *et al*. [13] was performed only on patients with successful PCI, so there are some differences in the patient groups. And our study considered the primary endpoint as MACCEs, but Ben-Yehuda *et al*. [8] and Lee *et al*. [13] only evaluated mortality, and the follow up period was long-term as 3 and 4.4 years, respectively. In Park* et al*. [23], as in the present study, at 1 year follow up, PMI did not affect the clinical outcome, but in the 3 year clinical outcome, adverse events increased statistically significantly. This suggests that further evaluation of long-term clinical outcome is needed in the present study population as well. But above all, in these previous studies, patients with PMI already had higher risk profiles such as higher number of implanted stents [8, 9, 13, 23], longer stent length [8, 9, 13, 21, 23], higher syntax score [8], LM disease [8, 9, 21, 23], MVD [9, 13, 23], and rotablator use [9] compared to patients without PMI. Thus, they could not determine whether PMI directly affected clinical outcome as a causal factor or merely reflected progressive CAD and procedure complexity as an indirect indicator. In our study, there was no difference in the above-mentioned high risk profiles between non-PMI and PMI groups. The two groups showed no statistically significant difference in clinical outcome. The result of the present study was consistent with a previous study on the impact of PMI in patients with chronic total occlusion (CTO) [12], another progressive CAD type such as severe CAC. One study using intravascular ultrasound also showed that patients developing PMI after PCI had more extensive atherosclerosis [5]. These findings and our results suggest that PMI is only a marker of advanced atherosclerosis, not a causal factor in clinical outcome.

## 5. Study Limitation

Our study had several limitations. First, this was a non-randomized, observational, and retrospective study with a possibility of selection bias. Second, when defining PMI, clinical sign and ECG corresponding to ancillary criteria were not considered. We only considered biomarker elevation. Howerever, when ancillary criteria are applied, the CK-MB threshold is lowered to >5× ULN, and in this case, it may not affect the clinical outcome, unlike applying the only >10× ULN [8]. Therefore, it seems that not applying ancillary criteria did not affect the study result. Third, other causes of CK-MB elevation were not evaluated or excluded. Finally, even though this study was multicenter study, the number of enrolled patients was not large enough because RA was an infrequently performed procedure. Therefore, modest statistical power was also a major limitation of this study. Therefore, caution is required when interpreting our results.

## 6. Conclusions

This study shows that PMI after RA in patients with severe CAC was associated with more frequent in-hospital events and a nonsignificant trend for more events during 1 year follow-up. These findings require confirmation in larger studies with longer follow-up.

## Data Availability

The datasets used and/or analyzed during the current study are available from the corresponding author on reasonable request.
